# Repertory of Foot-Sweat

**Published:** 1894-06

**Authors:** Olin M. Drake

**Affiliations:** Boston, Mass.


### REPERTORY OF FOOT-SWEAT.

OLIN M. DRAKE, M. D., BOSTON, MASS.

I have no doubt that many of my colleagues, among the true followers of Hahnemann, have often felt, like myself, the need of a complete repertory on *Foot-sweat.* The material for this repertory was culled from the materia medicas of Hahnemann, Allen, Hering, Lippe, Farrington, *Jahr's Manual, Jahr's and Possart's New Manual; Hull's Jahr, Hale, and Mure;* and from the periodical medical literature of our school in the English language for the past thirty years.

I have given the modalities and concomitants when I have found them in the materia medica—not otherwise, with a very few exceptions.

I do not believe I have omitted any Foot-sweat symptoms, but if I have overlooked any I would be glad to be informed of the fact.

I hope this short paper will be of use to some of my busy *confrères*, assisting them in finding the simillimum from the true Hahnemannian standpoint. This is my principal excuse in yielding to the wishes of several friends, who have requested me to send it to THE HOMŒOPATHIC PHYSICIAN for publication.

SWEAT OF THE FEET. Acon., Am-car., Am-m., Anan., Angu., Ars., Ars-m., Arun., Bar-c., Bell., Benz-ac., Bry., Calc-c. (Calc-p.), Cale., Cann-s., Canth., Carb-ac., Carb-an., Carb-v., Caus., Cenchris-c., Cham., Chlol., Cimic., Coc-c., Cocc., Coff., Coloc., Cup., Cyc., Dro., Euphm., Fago., Farfa., Fl-ac., Graph., Hæm., Hell., Hep., Hur. (Hype.), Ib., Ind., Iod., Ip., Jab., K-bi., K-ca., K-ph., Kalm., Kre., Lach., Lac-ac., Laur., Led., Lil-t., Lyc., Mag-m., Mang., Medor., Merc-sol., Merc-sulph., Mez., Mur-ac., Na-c., Na-m., Nit-ac., Nx-j., Ox-ac., Pb., Ped., Petrol., Phos., Pho-ac., Phyt., Pic-ac., Pod., Pso., Pul., Rhu-t., Sabi., Sal-ac., Sanic., Sec-c., Sep., Sil., Squ., Stap., Sul., Tel., Thea., Thu., Verat., Verat-v., Wies., Zn.

SWEAT OF THE FEET, CONSTANT. Sil., Thu.
— — — — MORNING. Euphm., Sul.
— — — — —, in bed. Bry., Lach., Merc-sol., Phos., Pul., Sabi.
— — — — —, after rising. Am-m.
— — — — P. M. Graph., Ple.
— — — — EVENING. Calc-c., Coc-c., Grap., Pod.
— — — — —, in bed. Calc., Cle., Mur-ac.
— — — — NIGHT. Coloc., Nit-ac., Sul., Thu.
— — — — —, on waking. Mang.
— — — — MENSES, before, during, and after. Calc-c.
— — — — —, during, from severity of pain. Verat.
— — — — SITTING, while. Bell.
— — — — — — in warm room. Mez.
— — — — SUMMER. Cham.
— — — — WINTER. Medor.
— — — — RIGHT FOOT, night. Sul.
— — — — — —, the left remaining quite dry. Ple.
— — — — LEFT foot. Cham., Nit-ac.
— — — —, with moist, painless vesicles between toes. Hell.
— — — — BURNING with, of feet. Calc-c., Calc., Lyc., Mur-ac., Petrol., Sep., Sul., Thu.
— — — — COLDNESS with, of the feet. Acon., Bell., Calc-c., Cann-s., Dro., Fago., Ib., Ind., Iod., Ip., Lyc., Pic-ac., Sil., Sul., Verat.
— — — — ITCHING with, of the soles. Sil., Sul.
— — — — SWELLING with, of the feet. Graph., Iod., K-ca., Kre., Lyc., Pb., Petrol., Pho-ac., Saba.
— — — — WALKING, when. Carb-v., Graph., Na-c.
— — — — PAIN, with tearing, in feet and hands. Graph.
— — — — SORENESS, with, at end of nails. Merc-sulph.
— — — — COLD. Acon., Ars., Benz-ac., Calc-c., Canth., Carb-v., Cimic., Cocc., Dro., Fago., Farfa., Hep., Hur., Ip., K-ph., Lil-t., Lyc., Medor., Merc-sol., Mez., Mur-ac., Nit-ac., Ox-ac., Pb., Ped., Squ., Stap., Sul., Verat-v.
— — — — — Moisture, rather than sweat. Calc-c.
— — — — — Diarrhœa, during. Sulph.
— — — — COLD, AND HANDS, in typhoid fever. Carb-v.

SWEAT OF THE FEET, COLD AND CLAMMY. Calc-c., Lau., Merc-sol., Pic-ac., Sanic., Sul., Thea.

— — — — — — — evenings. Pic-ac.

— — — — — one foot hot, the other cold. Hur., Lyc.

— — — — — and clammy, up to knees. Lau.

— — — — — — damp, followed by very cold feet. Ped.

— — — — — followed by very hot feet. Hur.

— — — — — and sticky. Calc-c.

— — — — WARM. Ars-m., Led.

— — — — EXCORIATING. Bar-c., Calc-c., Carb-v., Coff., Grap., Iod., Lyc., Nit-ac., Sec-c., Sanic., Sep., Sil., Zn.

— — — — — or corrosive, so much so that the hose and shoes are quickly destroyed. Sec-c.

— — — — — making feet raw or sore. Cham., Calc-c., Grap., Lyc., Nit-ac., Petrol., Saba., Squ., Zn.

— — — — — making soles raw or sore. Bar-c., Calc-c., Nit-ac., Petrol., Saba., Sil.

— — — — — causing soreness of soles, with sticking pains, as if walking on pins. Nit-ac.

— — — — — making toes raw or sore. Bar-c., Carb-v., Coff., Grap., Nit-ac., Sep., Sil., Zn.

— — — — FETID. Am-car., Am-m., Anan., Arun., Bar-c., Calc-c., Carb-ac., Chlol., Grap., K-ca., Kalm., Lac-ac., Lyc., Na-m., Nit-ac., Nx-j., Pb., Petrol., Pho., Pso., Rhu-t., Sal-ac., Sanic., Sec-c., Sep., Sil., Sul., Thu., Wies., Zn.

— — — — — moisture, rather than sweat. Petrol.

— — — — — Cheese, smelling like old. Pb.

— — — — — Eggs rotten, smelling like. Staph.

— — — — — Sole leather, smelling like. Cob.

— — — — — Sour smelling. Calc-c., Cob., Na-m.

— — — — — Urine, smelling like. Canth., Coloc.

— — — — — Menses, after. Sep., Sil.

— — — — — in persons of rheumatic tendency, much exposed to rough weather and hard labor. Rhu-t.

— — — — ODORLESS. Graph., Lac-ac., Merc-sol.

— — — — PROFUSE. Ars-m., Arun., Carb-an., Carb-v., Cenchris-c., Cham., Coloc., Fl-ac., Grap., Ind., K-ca.,

Kre., Lach., Lac-ac., Lyc., Nit-ac., Petrol., Phyt., Ple., Pul., Saba., Sal-ac., Sec-c., Sep., Sil., Stap., Sul., Thu., Zn.

SWEAT OF THE FEET, PROFUSE. Can almost wring the hose. Cenchris-c.

— — — — — Right foot of, so that the hose was completely soaked. Ple.

— — — — — obliged to change hose, which was wet through. Sul., Thu.

— — — — — the feet being cold in winter and sore in summer. Sil.

— — — SOLES. Acon., Am-m., Arn., Fago., K-ca., Merc-sol., Na-m., Nit-ac., Nx-m., Oxytropis, Pb., Petrol., Saba., Sanic., Sil., Sul., Wies.

— — — — soles always wet, and without feeling. Nx-m.

— — — — COLD, on the left. Sul.

— — — — as though he had stepped into cold water. Sanic.

— — — — FETID. Pb., Petrol., Sil.

— — — — — with tender feet. Petrol.

— — — — SOUR. Na-m.

— — — — STICKY, as though he had stepped into molasses. Sanic.

— — — — — the hose sticking to feet. Sanic.

— — — — with callosities on the soles, which are painful on walking. Bar-c.

— — — — causing exfoliation of skin of soles. Thu.

— — — HEELS, PROFUSE. Thu.

— — — TOES. Acon., Arn., Lach., Phyt., Pul., Sep., Squ., Tel., Thu., Zn.

— — — — mornings in bed. Lach.

— — — — walking when. Grap.

— — — — FETID, with redness and swelling of tips. Thu.

— — — — UNDER. Phyt., Tarax.

— — — — BETWEEN. Acon., Arn., Cle., Cob., Cyc., Ferr., K-ca., Lyc., Sanic., Sep., Sil., Squ., Tarax., Thu.

— — — — BETWEEN. Softens the corns, so that they can be taken out with the finger-nails. Lyc.

— — — — — Fetid. Cyc., Pul., Sil., Thu.

SWEAT OF THE FEET, SUPPRESSED. Apis, Ars. (Awa Samoa), Bad., Bar-c., Bar-m., Coch., Colch., Cup., Form., Hæm., K-ca., Na-m., Nit-ac., Nx-j., Pul., Rhu-t., Sep., Sil., Thuj. Zn.

— — — — — by a cold. Apis.

— — — — — — — — bath. Bar-c.

— — — — — — getting wet. Colch., Sil.

— — — — — followed by angina tonsillaris. Bar-c.

— — — — — — — chorea. Form.

— — — — — — — icy cold feet and legs, worse evenings in bed. Sil.

— — — — — — — eyes, affection of. Sil.

— — — — — — — lameness. Bar-c.

— — — — — — — lost appetite. Sil.

— — — — — — — nervous excitement. Zn.

— — — — — — — palpitation. Ars., Hæm.

— — — — — — — paralysis of feet. Zn.

— — — — — — — toothache. Sil.

FEET, FETID ODOR OF, WITHOUT SWEAT. Grap., Sep., Sil.

— — — — — — carrion like. Sil.

— — — — — — sour. Sil.

Sensation as though the feet were sweating, and the hose soaked full. Lac-ac.

— — — a cold sweat were trickling down the feet, at night on rising. Croc.

— — — had on cold damp stockings. Calc-c., Sep.

