# Correction

**Published:** 1894-07

**Authors:** 


CORRECTION: In the Repertory of Foot Sweat by Dr. Olin M. Drake in the June number the last line on page 175 should read:

"Sensation as though he had on cold damp stockings, Calc-c., Saponinum, *not* Sepia as printed.

